# Supplementary material for: Structural basis for activation, assembly and membrane binding of ESCRT-III Snf7 filaments
Source: eLife. 2015 Dec 15;4:e12548. doi: 10.7554/eLife.12548 (PMC4720517; doi:10.7554/eLife.12548)
Supplement: Supplementary file 1. — A list of plasmids for Saccharomyces cerevisiae expression, Escherichia coli expression for protein purification, and Saccharomyces cerevisiae strains. DOI: http://dx.doi.org/10.7554/eLife.12548.034 [file elife-12548-supp1.docx]

**Supplemental file 1, related to MATERIALS AND METHODS. Plasmids and Yeast Strains Used in This Study**

| **Plasmids for *Saccharomyces cerevisiae* Expression** | | | | | |
| --- | --- | --- | --- | --- | --- |
| **Plasmid** | | **Mutations** | **Reference** | | |
| pRS416 | | N/A | (Sikorski and Hieter, 1989) | | |
| pRS414 | | N/A | (Sikorski and Hieter, 1989) | | |
| pRS416-*SNF7* | | Wild-type | (Henne et al., 2012) | | |
| pRS414-*SNF7* | | Wild-type | this study | | |
| pRS416-*snf7*^Q90C^ | | Q90C | this study | | |
| pRS416-*snf7*^M130C^ | | M130C | this study | | |
| pRS416-*snf7*^Q90C M130C^ | | Q90C M130C | this study | | |
| pRS416-*snf7*^T20C^ | | T20C | this study | | |
| pRS416-*snf7*^K35C^ | | K35C | this study | | |
| pRS416-*snf7*^K60C^ | | K60C | this study | | |
| pRS416-*snf7*^E88C^ | | E88C | this study | | |
| pRS416-*snf7*^H118C^ | | H118C | this study | | |
| pRS416-*snf7*^G140C^ | | G140C | this study | | |
| pRS416-*snf7*^E88C H118C^ | | E88C H118C | this study | | |
| pRS416-*snf7*^K60C A66C^ | | K60C A66C | this study | | |
| pRS416-*snf7*^T83E^ | | T83E | this study | | |
| pRS416-*snf7*^M87E^ | | M87E | this study | | |
| pRS416-*snf7*^Q90K^ | | Q90K | this study | | |
| pRS416-*snf7*^I94E^ | | I94E | this study | | |
| pRS416-*snf7*^A97K^ | | A97K | this study | | |
| pRS416-*snf7*^L99K^ | | L99K | this study | | |
| pRS416-*snf7*^L101E^ | | L101E | this study | | |
| pRS416-*snf7*^T103E^ | | T103E | this study | | |
| pRS416-*snf7*^M104E^ | | M104E | this study | | |
| pRS416-*snf7*^M107E^ | | M107E | this study | | |
| pRS416-*snf7*^M114E^ | | M114E | this study | | |
| pRS416-*snf7*^I117E^ | | I117E | this study | | |
| pRS416-snf7^L121D^ | | L121D | (Saksena et al., 2009) | | |
| pRS416-*snf7*^R25E^ | | R25E | this study | | |
| pRS416-*snf7*^H29E^ | | H29E | this study | | |
| pRS416-*snf7*^K36E^ | | K36E | this study | | |
| pRS416-*snf7*^E95K^ | | E95K | this study | | |
| pRS416-*snf7*^E102K^ | | E102K | this study | | |
| pRS416-*snf7*^E109K^ | | E109K | this study | | |
| pRS414-*snf7*^R25E K36E^ | | R25E K36E | this study | | |
| pRS416-*snf7*^E95K E109K^ | | E95K E109K | this study | | |
| pRS414-*snf7*^R25E H29E K36E^ | | R25E H29E K36E | this study | | |
| pRS416-*snf7*^E95K E102K E109K^ | | E95K E102K E109K | this study | | |
| pRS414-*snf7*^K35C^ | | K35C | this study | | |
| pRS416-*snf7*^A63C^ | | A63C | this study | | |
| pRS416-*snf7*^K69C^ | | K69C | this study | | |
| pRS416-*snf7*^Q75C^ | | Q75C | this study | | |
| pRS416-*snf7*^E81C^ | | E81C | this study | | |
| pRS416-*snf7*^E95C^ | | E95C | this study | | |
| pRS416-*snf7*^E102C^ | | E102C | this study | | |
| pRS416-*snf7*^V126E^ | | V126E | this study | | |
| pRS416-*snf7*^M130E^ | | M130E | this study | | |
| pRS416-*snf7*^I133E^ | | I133E | this study | | |
| pRS416-*snf7*^A51E^ | | A51E | this study | | |
| pRS416-*snf7*^L55E^ | | L55E | this study | | |
| pRS416-*snf7*^L67E^ | | L67E | (Henne et al., 2012) | | |
| pRS416-*snf7*^N59P^ | | N59P | this study | | |
| pRS416-*snf7*^E102P^ | | E102P | this study | | |
| pRS416-*snf7*^L121P^ | | L121P | this study | | |
|  | |  |  | | |
| **Plasmids for *Escherichia coli* Expression for Protein Purification** | | | | | |
| **Plasmid** | | **Mutations** | | | **Reference** |
| pET28a-*SMT3-snf7*^12-150^ | | *SMT3*-*snf7*N12-P150 | | | this study |
| pET23d-*snf7*^R52E^ | | R52E | | | (Henne et al., 2012) |
| pET23d-*snf7*^R52E E88C H118C^ | | R52E E88C H118C | | | this study |
| pET23d-*snf7*^R52E E88C G140C^ | | R52E E88C G140C | | | this study |
| pET23d-*snf7*^R52E H118C G140C^ | | R52E H118C G140C | | | this study |
| pET23d-*snf7*^R52E T20C^ | | R52E T20C | | | this study |
| pET23d-*snf7*^R52E K35C^ | | R52E K35C | | | this study |
| pET23d-*snf7*^R52E K60C^ | | R52E K60C | | | this study |
| pET23d-*snf7*^R52E E88C^ | | R52E E88C | | | this study |
| pET23d-*snf7*^R52E H118C^ | | R52E H118C | | | this study |
| pET23d-*snf7*^R52E G140C^ | | R52E G140C | | | this study |
| pET23d-*snf7*^R52E K60C A66C^ | | R52E K60C A66C | | | this study |
| pET23d-*snf7*^R52E Q90K^ | | R52E Q90K | | | this study |
| pET23d-*snf7*^R52E I94E^ | | R52E I94E | | | this study |
| pET23d-*snf7*^R52E M107E^ | | R52E M107E | | | this study |
| pET23d-*snf7*^R52E M114E^ | | R52E M114E | | | this study |
| pET23d-*snf7*^R52E R25E H29E K36E^ | | R52E R25E H29E K36E | | | this study |
| pET23d-*snf7*^R52E E95K E102K E109K^ | | R52E E95K E102K E109K | | | this study |
| pET23d-*snf7*^R52E V126E^ | | R52E V126E | | | this study |
| pET23d-*snf7*^R52E I133E^ | | R52E I133E | | | this study |
| pET23d-*VPS24* | | Wild-type | | | (Henne et al., 2012) |
| pET23d-*VPS2* | | Wild-type | | | (Henne et al., 2012) |
|  | |  | | |  |
| **Yeast Strains Used in This Study** | | | | | |
| **Strain** | **Genotype** | | | **Reference** | |
| SEY6210.1 | *Mat a, leu2-3, 2-112, ura3-52, his3-*Δ*200, trp1-*Δ*901, lys2-801, suc2-*Δ*9* | | | (Robinson et al., 1988) | |
| MBY24 | SEY6210.1; *snf7*Δ*::HIS3* | | | (Babst et al., 2002) | |
| NBY44 | SEY6210.1; *snf7*Δ*::HIS3; MUP1-PHLOURIN::KAN* | | | (Henne et al., 2012) | |
